# Making the Shift from Research to Commercial Orchards: A Case Study in Aphid–Peach Tree Interactions as Affected by Nitrogen and Water Supplies

**DOI:** 10.3390/insects12111003

**Published:** 2021-11-08

**Authors:** Marie-Odile Jordan, Bruno Hucbourg, Aurore Drevet

**Affiliations:** 1 INRAE, UR1115 Plantes et Systèmes de Culture Horticoles (PSH), Domaine Saint-Paul, Site Agroparc, CEDEX 09, 84914 Avignon, France; 2 GRCETA de Basse Durance, 2 Route de Mollèges, CEDEX 02, 13210 Saint Rémy de Provence, France; bruno.hucbourg@grceta.fr (B.H.); aurore.drevet@grceta.fr (A.D.)

**Keywords:** *Myzus persicae*, *Myzus varians*, *Hyalopterus pruni*, *Prunus persica*, shoot development, shoot composition, infestation severity

## Abstract

**Simple Summary:**

Commercial orchards are amongst the most intensively sprayed crops, and alternative methods have to be found to replace pesticides. Limiting water and nitrogen (N) supply has shown to be effective in reducing aphid infestations under controlled conditions. To evaluate how far these techniques could be transferred to orchards subject to production constraints, an experiment was performed in a commercial orchard planted with two varieties differing in precocity and vigour. Limiting supplies of both water and N to trees was shown to reduce the severity of aphid infestation (green peach aphid, mealy plum aphid, and leaf curl aphid), although reducing only water supply was less effective. At shoot level, the composition and development of the infested shoots were only slightly affected by treatment, thereby indicating that aphids colonize shoots of similar condition, whose numbers are modulated by nutrition treatments. These results were consistent with variety and year. Limiting water and N supplies contributes not only to the control of aphid infestations, but also reduces nitrate leaching and the use of water, the consumption of which will inevitably need to a decrease due to climate change. However, the efficiency of aphid control could be enhanced by complementing these practices by other techniques such as adapted pruning or changes to ground cover.

**Abstract:**

Peach orchards are intensively sprayed crops, and alternative methods must be found to replace pesticides. We intend here to evaluate if limiting water and nitrogen (N) supply could be effective in controlling aphid infestation in commercial orchards. N and water supply were therefore either unrestricted or restricted by 30% only for water, or for both water and N, in 2018 and 2019 on trees of two contrasting varieties. Natural infestations (green peach aphid, mealy plum aphid, leaf curl aphid) were monitored regularly at tree and shoot level. Infested and control shoots were compared for their development during the infestation period, their apex concentrations of total N, amino acids, non-structural carbohydrates, and polyphenols at infestation peak. At tree level, limiting both water and N supplies decreased the proportion of infested shoots by 30%, and the number of trees hosting the most harmful specie by 20 to 50%. Limiting only N supplies had almost no effect on infestation severity. At shoot level, the apex N concentration of infested shoots was stable (around 3.2% dry weight) and was found to be independent of treatment, variety, and year. The remaining biochemical variables were not affected by infestation status but by variety and year. Shoot development was only slightly affected by treatment. Aphids colonized the most vigorous shoots, being those with longer apical ramifications in 2018 and higher growth rates in 2019, in comparison with the controls. The differences were, respectively, 40 and 55%. It was concluded that a double restriction in water and N could limit, but not control, aphid infestations in commercial orchards.

## 1. Introduction

Commercial orchards are among the most intensively sprayed crops. However, pest control using chemicals is environmentally costly and becomes increasingly difficult due the development of resistance to the most commonly used pesticides [1]. Production sustainability should thus be enhanced by using alternative methods to the use of pesticides [2,3]. Among them, biological control tends to reduce pest pressure by using various techniques, such as the fostering of natural enemies (predators, parasitoids), spraying with natural pesticides, and establishing wild strips or companion plants. Other methods focus more on the plants, attempting to breed resistant cultivars or adapting current cultural practices (e.g., fertilization, irrigation or pruning) to reduce plant susceptibility to pests, by fostering plant traits which are unfavourable to pests (e.g., production of defence compounds, plant shaping to limit pest sheltering and dissemination within the plant, reduced access to food resources, among others). The effectiveness of cultural practices relies on the existence of cross-tolerance between biotic and abiotic stresses [4], and on the dependence of pest and plant development [5]. This aspect has however only been little investigated, and prior to its implementation in commercial orchards, the main determinants of plant susceptibility have to be identified, and their variations together with growing conditions, genotype, and tree life background characterized.

Of high economic importance, the peach tree–aphid system has been one of the most studied in recent years. Thus, peach (*Prunus persica* L. Basch) ranks first in stone fruit production, both worldwide (25.7 million tons) [6] and in the Mediterranean basin (3.8 million tons) [6]. Of the numerous herbivore pests, the green peach aphid (*Myzus persicae* Sulzer) represents a major threat that affects fruit production for several years and transmits viruses. Peach susceptibility to green peach aphid has been found to be positively related to shoot development (emergence of new organs) [7,8] and growth (increase in organ size) [5], as well as to apex concentrations [8,9,10,11] of total N (up to a threshold level), amino acids and non-structural sugars. The polyphenol concentrations, in contrast, were negatively related to tree susceptibility [8,12]. Tree susceptibility is thus determined by developmental and trophic aspects, i.e., by the balance between plant or shoot development, growth, and composition. Growing conditions and tree life background affect each of these aspects differently, with possible antagonistic effects on the defence capability. To avoid producing contradictory results, our understanding of plant pest interactions has to therefore consider simultaneously all these aspects.

For practical and feasibility reasons, studies on integrated plant functioning have mostly been performed on small, young, potted trees grown under controlled, or semi-controlled, conditions. However, aging and orchard conditions affect different aspects of plant metabolism. So, on adult trees, fruits represent a principal trophic sink in competition with vegetative development [13,14] when aphid infestation could arise. The relative size of the nitrogen (N) and carbon (C) reservoirs (i.e., the proportion of the roots and the proportion of one-year-old wood), increase also with plant age, meaning that store mobilization could buffer limited supplies over long periods (i.e., several years) [15]. This could, for instance, explain why defoliation or foliar damage, whatever their cause, are less harmful on adult than on young trees. More generally, tree growth modifies the balance between shoot or tree size, shape, and composition. However, not all determinants of plant susceptibility are affected similarly, thereby modifying their effects on plant-pest interactions.

The results obtained under semi-controlled conditions must therefore be adapted to productive trees grown in open fields. The present study focuses on the peach tree—aphid system. The optimal values (e.g., rates, dimensions, concentrations) of the key plant variables determined in previous studies [3,5,8] will be re-evaluated under orchard conditions, and their final impact on aphid infestations analysed, considering the functional dependence between organ formation, growth, and composition. To reach this goal, an experiment was carried out under contrasting production conditions, in an organic commercial orchard regularly subject to severe aphid infestations. Reference trees were selected from two genotypes of different precocity and vigour established on adjacent plots, and then received different levels of water and nitrogen supplies. The treatments were designed to be consistent with regional agricultural practice. They were repeated over two consecutive years, during which we were reliant on natural aphid infestations, artificial ones not being feasible in commercial orchards. This represented a major constraint, which led us to consider not only the green peach aphid but also to include the two additional species present, (i) the leaf-curl plum aphid (*Myzus varians* Davidson) and (ii) the mealy plum aphid (*Hyalopterus pruni* Geoffer). Thus, we assumed that the determinisms of plant susceptibility to green peach aphids and to both additional species were mostly similar, since they developed at the same times and on the same organs as the green peach aphid, sometimes in mixed colonies. Another assumption behind this work is that the external environmental aphid pressure was similar for all selected trees, meaning that aphids settled on the ones best able to foster their development.

This study aims first to discuss the effects of treatment, year, and variety on severity of infestation, defined as the proportion of infested shoots, and then to explain these effects, comparing the overall condition of infested and non-infested shoots. Indeed, aphid dispersion within the canopy is related to the number of shoots whose functional balance enables the establishment of new colonies, which may vary with cultural practices, genotype, or pedo-climate. Infested and non-infested (i.e., control) shoots were therefore compared for their growth, analysed from an architectural point of view, as well as their composition in non-structural carbohydrates (NSC), total N, amino acids, and polyphenol content. At shoot level, this extensive screening allowed (i) the defining of conditions which favoured (or prevented) aphid infestation, (ii) the determining to what extent these conditions depended on cultural practices (irrigation and fertilization), genotype or year, and (iii) the identifying of differences from those previously established in controlled or semi-controlled conditions. At tree level, this study also gives insight into how infestation severity may be manipulated by routine cultural practices.

## 2. Materials and Methods

### 2.1. Experimental Design

The study was carried out over two consecutive years (2018 and 2019), in a 1.5 ha organic commercial orchard located near Avignon (Southeast France, Lat: 43.89°, Long: 4.77°), established in 2008, at a density of 555 trees/ha (6 m and 3 m distances between rows and between trees, respectively), trained in a “gobelet” or open-centre shape. The rows, oriented E-W, were protected from dominant north winds by cypress hedges. The orchard is composed of two varieties of peach trees, both susceptible to aphids: Ivoire (vigorous, early ripening white peach) and Conquise (less vigorous, middle early ripening, white peach), grafted onto Cadaman-Avimag. This rootstock is adapted to the deep, clay-silty, and occasionally (especially in winter) anoxic soil. The aphicide treatment consisted of a single spray of mineral oil during winter. It was, similar to all cultural operations (pruning, thinning, harvesting, interrow mowing, prophylactic treatments), coherent with regional standards, prescribed (date, dose or intensity) and achieved by the orchard owner. The experimental treatments were applied to preselected trees. This treatment consisted of three modalities: the water and N inputs were either (i) unrestricted, i.e., those applied by the orchard owner (control or HN treatment), (ii) restricted only in water (hN treatment), or (iii) restricted in both water and N (hn treatment). The water supply was limited after commencement of irrigation (June 19 in 2018, June 1 in 2019) by reducing (30 L h^−1^ vs. 45 L h^−1^) the flow rate of the irrigation drippers. For N, both the 2018 and 2019 inputs were reduced. As a result, each HN tree received, from irrigation start to July 25 (i.e., until the end of harvest), 22 L of water in 2018 and 23 L in 2019, which had to be added to the rainfall (86 and 48 mm between April 1 and July 25 in 2018 and 2019, respectively). The N supplies were of 84 and 83 g N in 2018 and 2019, respectively. For the restricted treatments, the inputs were reduced by 30% as detailed in Table 1.

The 54 selected trees were distributed in 18 groups (nine per variety) of three adjacent individuals, chosen for their homogeneity in terms of vigour, i.e., trunk diameter (53 cm ± 1.2 SE—standard error—for Ivoire, 58 cm ± 0.9 for Conquise) and number of branches 113 ± 4 for Ivoire and 115 9 ± 4 for Conquise. The 18 groups were located along six rows (i.e., three rows per variety, three groups per row). Each treatment modality was applied to three groups (i.e., nine trees) per variety: one per row, and the position of each modality along the row was varied depending on the row (e.g., from the nearest tree group on the first row, to the median group on the second row, and then to the furthest one on the third row). Each group received the same treatment in 2018 and 2019.

### 2.2. In Situ Measurements

The air temperature, rainfall and soil humidity were recorded every 15 min with an automated weather station and four capacitive sensors positioned at 40 cm soil depth, to regulate the irrigation and to record any possible extreme climatic events (frosts, etc.).

For each tree, we selected five (2018) and seven (2019) unbranched and one-year-old shoots at infestation start (i.e., before 3 May in 2018 and 9 May in 2019). Two of them were infested when selected, and the remaining (three in 2018 and five in 2019) were aphid-free controls of similar size and shape to the infested ones. Their growth was then monitored on an architectural basis, each shoot being considered as a collection of growth units (or GU) comprising an internode and its upper node with the attached leaf and axillary bud [16]. Each axillary bud present on the selected shoots can, or cannot, give rise to a long daughter axis. The number of their expanded leaves corresponds to the number of their fully developed GUs. They were counted on the selected shoot stems and on each of their long daughter axes, previously positioned along the parent shoots by the rank of their father’s GU. Therefore, the number of leaves on a selected shoot GU was correlated to the length of the daughter to which it gave rise, if any, and was equal to one (the shoot father’s GU leaf) in absence of ramification. The repartition of the leaves along the shoot stem was thus representative of the ramification process (in terms of numbers, positions, and lengths). The measurements were taken at shoot selection, and then after fruit harvest during the period of shoot growth rest (see discussion), i.e., on 25 July 2018, and 11 July 2019.

### 2.3. Aphid Pressure in the Orchard

The same observer monitored the aphid infestation fortnightly in 2018 (i.e., between 30 April and 3 July) and weekly in 2019 (i.e., between 7 May and 30 June). The aphid species present on each selected tree and shoot was identified and the infestation status evaluated at tree and shoot level. More precisely, a class was assigned to each tree as follows: S0 (no aphid), S1 (less than 15% infested shoots), S2 (between 15 and 50%) and S3 (more than 50%). The shoots were classified as infested or controls according to the presence or absence of reproducing aphids. Indeed, single adults could be found on a shoot on one date but did not settle, i.e., were no longer present at the following count, thus indicating that aphids choose their hosting shoots by roaming within the canopy until they find a suitable site. The control shoots which became infested during the observation period were excluded from the statistical analyses.

Most trees were infested by more than one species. The dominant one was designated considering its abundance and harmfulness (green peach aphid > mealy plum aphid > leaf curl plum aphid). More precisely, a species was dominant at tree level if (i) it was more harmful and abundant, or (ii) it was more harmful and less abundant, but with a difference of only one class (e.g., S2 instead of S3, or S1 instead of S2). This rule was established following discussion with the orchard owner, considering his perception of the damage and economic losses due to aphid infestation. Different species could also be observed on the same shoot, all of them being passive phloem feeders which select their hosting shoot according to the same criteria.

### 2.4. Biochemical Determinations

The shoots were sampled on 16 May 2018, and 13 June 2019, i.e., during the period of rapid increase in the populations of the dominant aphid species. As far as possible, two samples were collected per tree, each of them ideally being composed of four to six growing apices (comprising the terminal meristems and the smallest unfolded leaves) of just emerging daughter axes inserted either on infested or on control shoots. It should be noted that on infested shoots, it was sometimes difficult to find apices young enough not to be colonized. The samples were immediately immersed in liquid N, and then stored at −80 °C until freeze-dried and ground in ball mills (MM301, Retsch, Germany) cooled with liquid N. The biochemical determinations were performed as described precisely in Jordan et al. [8]: (i) the total N using an elemental analyser (Flash EA 1112, Thermo Finnigan, Milan, Italy), (ii) starch and soluble sugars using an enzymatic method, (iii) amino acids by HPLC (Cortecs C18 column 4.6 mm × 150 mm, particle size 2.7 μm; Waters) in line with a 2475 multiwavelength fluorescence detector, and (iv) the polyphenols by HPLC (Uptisphere HDO column; Interchim, Montluçon, France) in line with a DAD UV–visible detector (Surveyor, Thermo Fisher Scientific, River Oaks Pkwy, SJ, USA). For the polyphenols, the measurements were made at two wavelengths (280 or 330 nm) and eight compounds (listed in Table 2) were identified by their standards. Four unidentified significant polyphenols peaks, resembling hydroxycinnamate derivatives, were also quantified using the 5CQA (Table 2) calibration curve.

### 2.5. Data Analysis

The statistical analyses were focused on shoot level and were performed using R software (R Core Team 2018, Vienna, Austria) [17].

Shoot growth was approximated by the leaf expansion rate (i.e., the increase in the number of fully developed GUs on the daughter axes and the selected shoot terminal bud). This rate was calculated for each shoot and corresponded to the daily increase in the number of expanded leaves during the observation period, divided by the length of the selected shoot stem (in meters). The C pools were quantified by considering either only the soluble sugars (glucose, sucrose, fructose, and sorbitol), i.e., the immediately available and transportable C forms, and the NSC pool, which includes also starch, i.e., the long-term storage form. The amino acids and polyphenols were summed to build the polyphenol and amino acid pools, but since each compound plays a specific role in plant metabolism, they were also considered separately in the analyses.

The leaf expansion rates, and the apex compositions were compared using non-parametric permutation tests performed at a 5% level, for which the empirical distributions of each variable effect were derived from 2500 random assignments of the tested dataset. The impact of treatments on each subgroup of shoots of similar infestation status, variety, and year was evaluated first. The absence of effect allowed the merging of shoots whatever their treatment, and to obtain pairwise comparisons between these newly formed groups, to assess, depending on the comparison, the effects of infestation status, year, or variety.

Kolmogorov–Smirnov tests were used following the previously described protocol, to evaluate how far the shoots differed: (i) in the distribution of their expanded leaves (i.e., of their long daughter axes) along the selected shoot stems, and (ii) in their apex’s amino acid, soluble sugar, and phenol profiles, i.e., in the respective contribution of each component to the cited pools.

## 3. Results

### 3.1. Aphid Pressure at Tree Level Varies More with Genotype and Year Than with Treatment

Aphid pressure in the orchard was assessed, evaluating for each selected tree, the dominant aphid species, and its abundance at infestation peak, i.e., the highest infestation severity recorded throughout the observation period (Table 3). These effects were lower in the trees of Conquise than of Ivoire for all treatments and years.

The effects of treatment varied over time. In 2018, the hn treatment, limited in water and N, comprised the lowest number of trees infested by the most harmful species, i.e., by the green peach aphid (5 and 4 for Ivoire and Conquise, respectively, vs. 8 and 5 for the HN treatment), and the highest number of aphid-free trees for both varieties (3 and 2 for Ivoire and Conquise, respectively, vs. 0 and 1 for the HN treatment). The aphid abundances were also the lowest on these trees. The differences in aphid species and abundances between the HN and hN treatments were small but the hN trees seemed to be slightly more susceptible to aphids for both varieties. In 2019, Conquise followed almost the same pattern as in 2018, since the leaf-curl plum aphid, i.e., the less harmful species, was overrepresented on the hN and hn trees (6 and 7 trees, respectively), subjected to deficit irrigation, compared to the HN ones (3 trees). This was especially true for the hn trees, which also had the smallest proportion of infested shoots (since all trees had fewer than 15% infested shoots vs. 7 trees for the HN treatment). It was again difficult to rank the two well fertilized treatments, since two of the nine HN trees were aphid-free and two others hosted the green peach aphid, vs. 0/9 and 1/9 hN trees, respectively. For Ivoire, one in three HN and hn trees were infested by leaf curl plum aphid. In contrast, the green peach aphid was overrepresented in the hN trees (7/9 trees), showing this treatment to be the most susceptible one.

Whatever the treatment and variety, the infestations were less detrimental in 2019 than in 2018. Indeed, the trees were more tolerant to the species which developed in the second year in almost all situations. This higher tolerance compensated widely for the slightly greater aphid abundances observed in 2019.

In short, the hN treatment was suspected to be the most favourable to aphids, but this assertion was evident only for Ivoire_2019. In contrast, the hn treatment was the less susceptible in all cases, except for Ivoire_2019. The effectiveness of treatment was however small compared to those of variety and year.

### 3.2. The Interactions between Shoot Growth and Infestation Depend on Year

**Figure 1:**
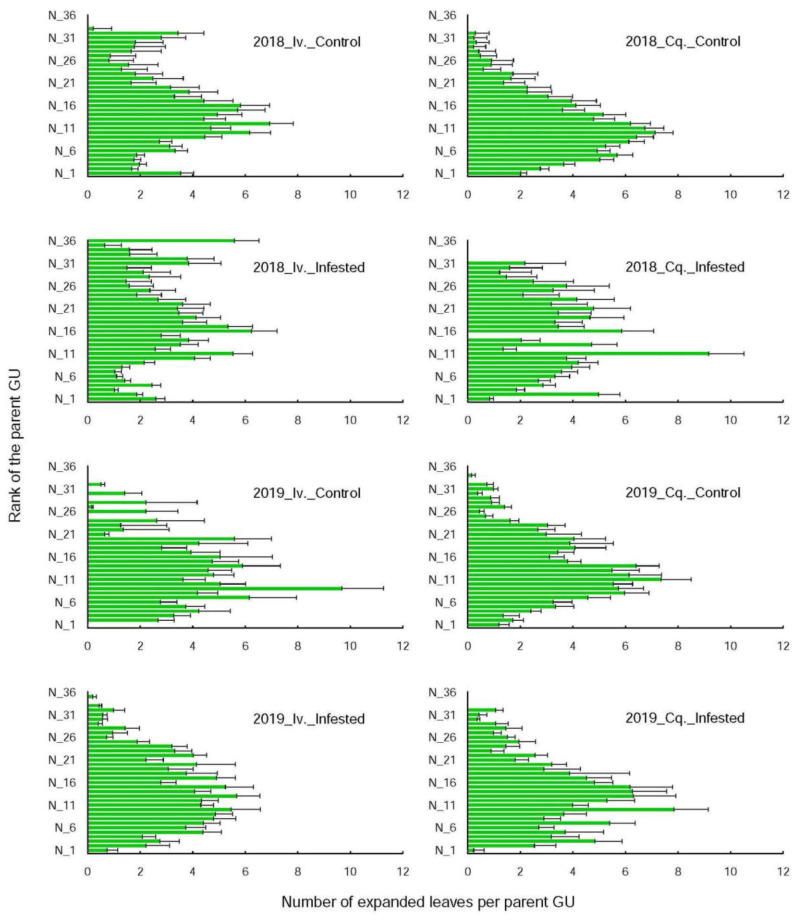
Repartition of the leaves along the shoots whose constitutive nodes were ranked from base Table 1. to N_36 for the two varieties (Iv. for Ivoire and Cq. for Conquise) and years (2018 and 2019). The bars stand for the mean % of leaves inserted on each node including those of their long daughter axes on the last counting date: 25 July 2018 and 11 July 2019 (i.e., after fruit harvest and during growth rest), and the lines for standard errors. No differences (Kolmogorov–Smirnov test, 5% level) could be found among treatments which were therefore grouped together.

Treatment and tree infestation severity affected neither shoot growth rates (i.e., the daily increase in the number of expanded leaves per shoot including those inserted on the long daughter axes), nor shoot shape (i.e., the repartition of those leaves along the shoot stem that depend on the number, positions, and lengths of the daughter axes). The growth rates (Table 4) on the infested and control shoots were however significantly (i.e., between 1.5 and 5.5 times) lower (i) in 2019 than in 2018 for both varieties. Conquise, known to be less vigorous, also exhibited growth rates whose values were lower by 25 to 65% than those of Ivoire. In contrast, the shoot shapes (Figure 1) were affected neither by variety, nor by year with one exception. For Ivoire, the leaves were more numerous on the upper growth units on the infested shoots in 2018 than in 2019. That meant that the daughter axes on those Ivoire infested shoots were not only more numerous (1.07 ± 0.3 vs. 0.96 ± 0.06) but also longer (18.5 ± 3.3 vs. 15.3 ± 1.1 leaves) in 2018 than in 2019.

The interactions between aphid development, shoot vigour and shape were year dependent. In 2018, the infested and control shoots had similar growth rates (Table 3), but the infested shoots bore more leaves on their upper growth units than the control ones. The difference was however significant only for Conquise, since the proportion of leaves inserted on the parent axes upper third were, respectively 14% and 4% for the infested and control shoots. In 2019, by contrast, the shoot growth rates were 1.7 (Ivoire) and 3 (Conquise) times higher for the infested shoots than for the controls, without any effect on shoot shape. The newly formed leaves (i.e., the leaves which expanded on the daughter axes during the aphid infestation period) were thus similarly distributed along the control and the infested shoots, despite being more numerous on the infested shoots.

### 3.3. The Relation between Infestation and Apex Composition Varied with Variety and Year

**Figure 2:**
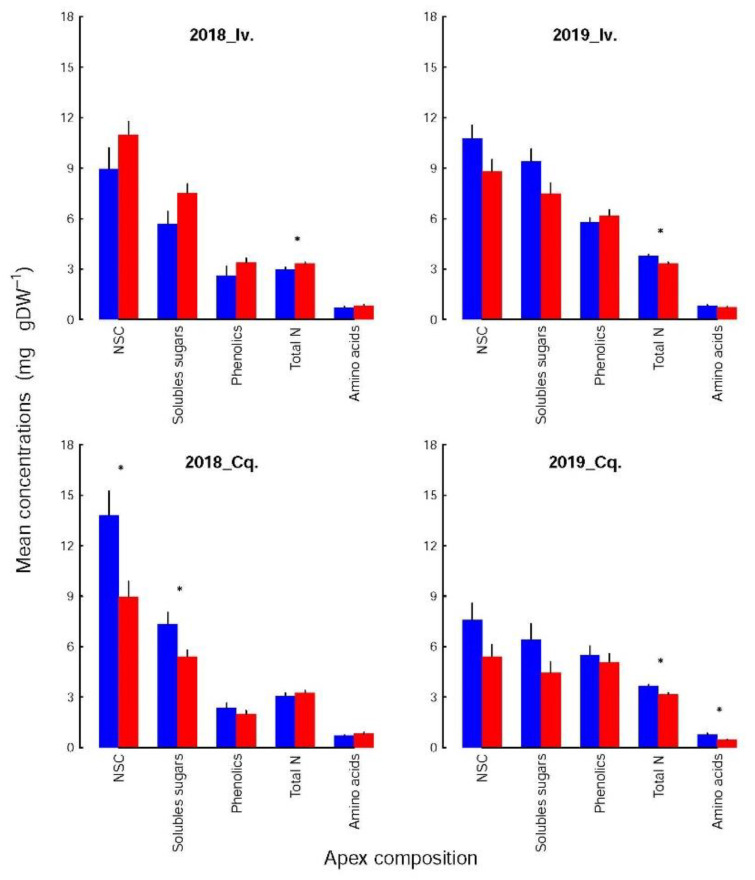
Mean concentrations (in % DW) of non-structural carbohydrates (NSC), soluble sugars, polyphenols (see Table 1 for details), total N and amino acids of infested (red bars) and control (blue bars) apices for the two varieties (Iv. for Ivoire and Cq. for Conquise) and years (2018 and 2019). The black lines represent standard errors. The groups whose concentrations differed significantly (randomization tests based on the generation of 2500 random orders) between the infested and control shoots are identified by asterisks. No differences could be found among treatments which were therefore grouped together.

Each biochemical family (Figure 2) was not affected to the same extent by variety and shoot infestation status. For the soluble sugars and NSC, the concentrations were higher in the control than in the infested shoots, except for Ivoire_2018. The differences were significant only for Conquise_2018 (13.8 ± 1.4 vs. 8.9 ± 0.9% DW for the NSC and 7.3 ± 0.71 vs. 5.4 ± 0.40%DW for the soluble sugars). The specific behaviour of Ivoire_2018 was related to the low concentration of soluble sugars in the controls (5.7% DW vs. more than 7.5% DW for the other groups of Ivoire shoots). These control shoots of Ivoire_2018 were subsequently the only group whose soluble sugar concentrations (i) were not significantly higher for Ivoire than for Conquise and (ii) significantly lower in 2018 than in 2019. The concentrations of soluble sugars (SolS_conc_) were also proportional to those of NSC (NSC_conc_), and the relations between both compounds (Equations (1) and (2), with R^2^_adj_ significance level: *** = 0.001) were affected neither by variety nor infestation status, but by year. These interannual fluctuations were due to a dramatic decrease (by 60% at least) in starch concentrations in 2019 compared to 2018.

**2018**: NSC_conc_ = 1.66 × SolS_conc_ + 1.66          R^2^_adj_ = 0.79 *** (1)

**2019**: NSC_conc_ = 1.08 × SolS_conc_ + 0.67          R^2^_adj_ = 0.98 *** (2)

For amino acids and total N, the concentrations (AA_conc_ and N_conc_) were lower in the infested shoots than in the controls in 2018, and higher in 2019. For total N, they varied between 3.16 and 3.32% DW in the infested shoots, whatever the variety and year. The concentrations in the control shoots were beyond this range in 2018, and above it in 2019. Significant differences could thus be evidenced (i) between the infested and control shoots except for Conquise_2018, (ii) between years for the control shoots but not for the infested ones, but (iii) not between varieties. The amino acids followed the same patterns, since they are linked to the total N by the following polynomial relation (Equation (3) with R^2^_adj_ significance level: ** = 0.01).

AA_conc_ = N_conc_^1.33^ − 1.55 × N_conc_ + 0.93          R^2^_adj_ = 0.22 ** (3)

**Figure 3:**
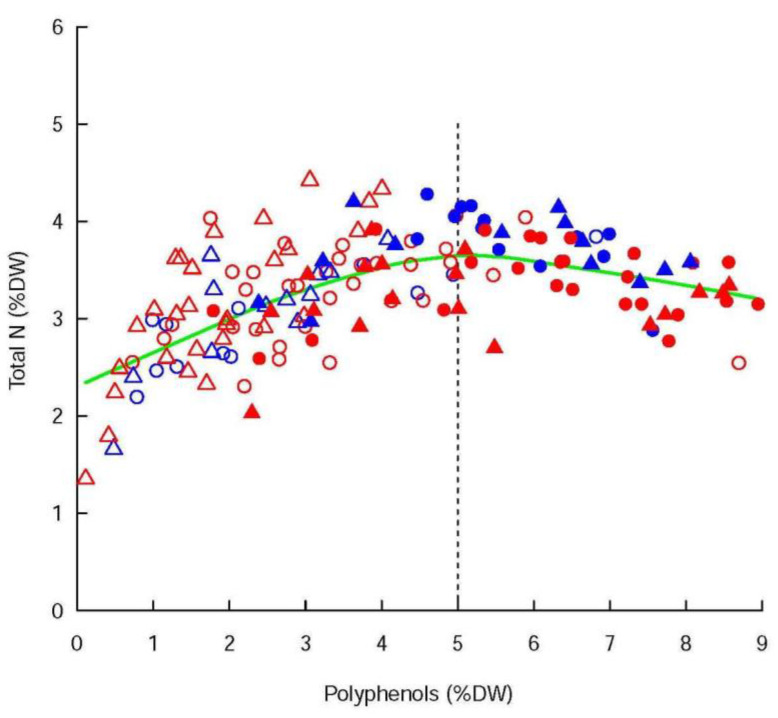
Relation between the concentrations (in %DW) of polyphenols and total N. The circles stand for the Ivoire shoots and the triangles for the Conquise ones. The infested shoots are in red, the control ones in blue. Empty symbols correspond to 2018 and full symbols to 2019. The green line was the result of scatterplot smoothing by locally weighted regression (LOWESS function in R). The following polynomial equations (Equations (4) and (5) with R^2^_adj_ significance level: *** = 0.001), which differ only in their final constant, fitted the ascending and descending parts of the smoothing curve. The vertical black line separates the data depending on whether Phe_conc_ is below or above the inversion point (Phe_conc_ = 5).

The polyphenols were not significantly affected by the infestation status, even if the concentrations were higher in the infested shoots than in the controls for Ivoire, and lower for Conquise. The difference among varieties were thus limited to the infested shoot. Indeed, in 2018 the concentrations of the infested shoots were of 3.38 ± 0.26% DW and 2.0 ± 0.20% DW for Ivoire and Conquise, respectively. In 2019 those values jumped to 6.16 ± 0.36% DW and 5.08 ± 0.49% DW, respectively. The greatest variation range was observed between years, since the concentrations, at least doubled between 2018 and 2019. Furthermore, the concentrations of polyphenols (Phe_conc_) and total N were linked (Figure 3): Phe_conc_ increased along with N_conc_ until a threshold corresponding to Phe_conc_ = 5% DW, then decreased slightly. Most of the values were below this threshold in 2018 and above it in 2019, and both families were consequently related positively in 2018 but negatively in 2019.

**Pheconc < 5% DW**:    N_conc_ = Phe_conc_^0.98^ − 0.93 × Phe_conc_ + 2.14    R^2^_adj_ = 0.51 *** (4)

**Pheconc > 5% DW**:    N_conc_ = Phe_conc_^0.98^ − 0.93 × Phe_conc_ + 3.23    R^2^_adj_ = 0.66 *** (5)

### 3.4. The Composition of the Different Families Differed Only by a Few Components

**Figure 4:**
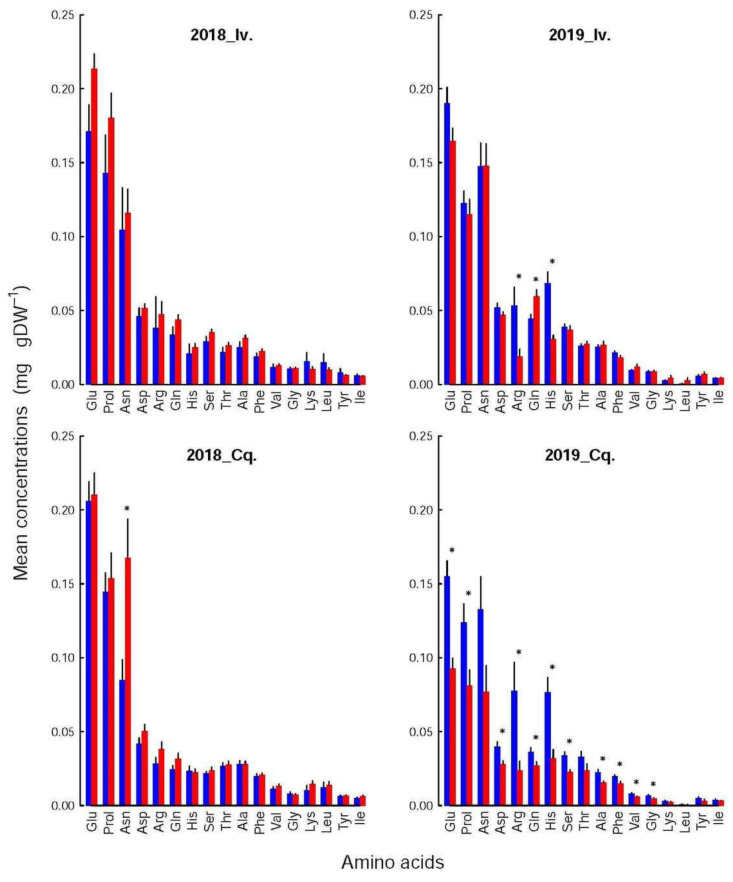
Mean concentrations (in ‰ DW) of amino acids of infested (red bars) and control, (blue bars) apices for the two varieties (Iv. for Ivoire and Cq. for Conquise) and years (2018 and 2019). The black lines represent standard errors. The groups whose concentrations differed significantly (randomization tests based on the generation of 2500 random orders) between the infested and control shoots are identified by asterisks. No differences could be found among treatments, which were therefore grouped together.

**Figure 5:**
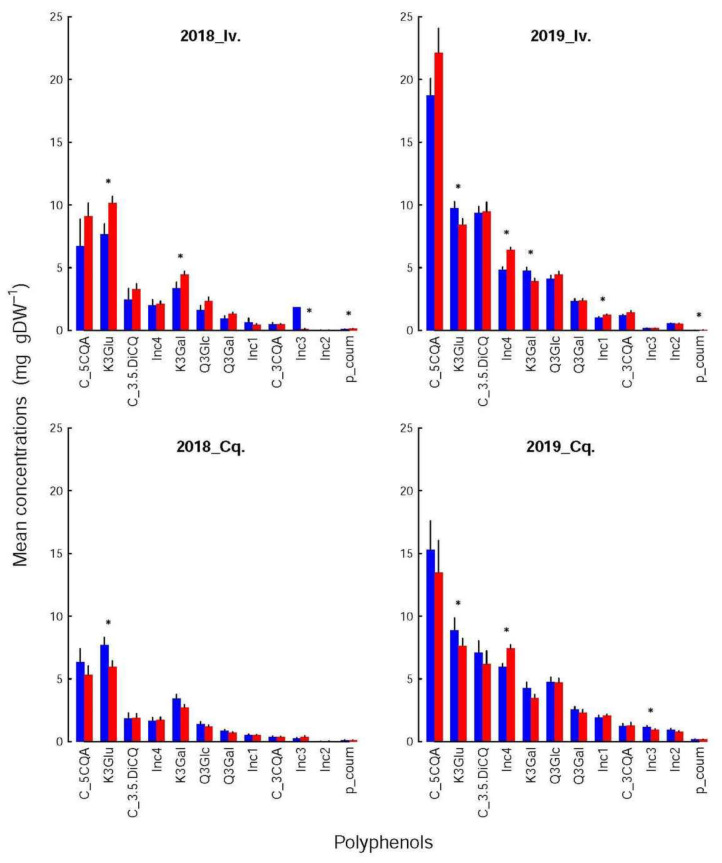
Mean concentrations (in ‰ DW) of polyphenols in infested (red bars) and control (blue bars) apices for the two varieties (Iv. for Ivoire and Cq. for Conquise) and years (2018 and 2019). The black lines represent standard errors. The groups whose concentrations differed significantly (randomization tests based on the generation of 2500 random orders) between the infested and control shoots are identified by asterisks. No differences could be found among treatments, which were therefore grouped together.

The composition (i.e., the relative contribution of each constituent) of the amino acids and the polyphenol pools were not altered by the shoot infestation status, year, or variety (Kolmogorov-Smirnoff tests), although the concentrations of several of their constituents could vary significantly. These variations were, however, too small to significantly affect the pool compositions. For instance, the relative contributions of each amino acid or polyphenol (Figure 4 and Figure 5) to its respective pool varied at most by 5% with shoot infestation status. The mean variation was 1.23 ± 0.12% but reached 2.08 ± 0.33% when the calculations were restricted to the compounds whose concentrations differed significantly between both groups. The greatest difference was observed for the NSC, more precisely for starch in Conquise_2018, whose contribution to the pool varied by 12%. That again did not affect the composition, but solely the concentrations of the NSC pool (Figure 3).

The differences between infested and control shoots depended more on variety and year. Thus, among the amino acids (Figure 4), only one compound was significantly affected in 2018 (Asn for Conquise), while in 2019, their number increased to three for Ivoire (His, Gln and Arg) and to 11 for Conquise (all acids but Asn, Thr, lys, Leu, Tyr, Ile). Concerning the polyphenols (Figure 5), the differences could either be found in all shoot groups (K3Glu), be restricted to Ivoire (p-coum), or be erratic (Inc3 and K3Gal for Ivoire_2018, Inc1 and Inc2 for Ivoire_2019, and Inc3 and Inc4 for Conquise_2019).

If dependent on variety (Table 4), the concentrations were higher for Ivoire than for Conquise for all compounds, but only some polyphenols. These exceptions were more restricted to the infested shoots and concerned only five compounds: p-coum, Inc1, Inc2, Inc3 and Inc4. All of them except Inc4 were minor compounds, i.e., contributed only little to the pool. All variations (increase or decrease) were in most cases limited to around 25%. They were around 40% for a few amino acids and reached 80% for two minor compounds: Leu and Inc3.

Furthermore, the concentrations of all the polyphenols, except for K3Gal, K3Glu and p-coum, were higher in 2019 than in 2018, thus explaining the huge interannual variation of the pool. Indeed, the concentrations increased by a factor of at least two to three for almost all compounds with two exceptions. For K3Glu and 3Gal they were limited to around 20%. The situation was more varied for the amino acids. The concentrations were higher by around 25% in 2019 than in 2018 for Ser in the control shoots for both varieties, Gln and Arg in the control shoots of Conquise, and Gln in the infested shoots of Ivoire. For His, the increase in the control shoots was higher: they doubled. In all other situations, except for Lys and Leu, the concentrations remained stable and decreased by at least 20% over the years. This decrease was higher for Lys and Leu (two minor acids): it was more important, being around 90%. The inter-annual difference therefore was small, and significant only for the infested shoots of Conquise.

## 4. Discussion

### 4.1. Dealing with the Inherent Complexity of Working in Commercial Orchards

Studies in commercial orchards have of necessity to be subject to specific constraints. Firstly, the design and application of the cultural practices (timing, intensity, delivery mode) remain the responsibility of the orchard owner. The proposed treatments must form part of the scheduled operations, and their degree must be consistent with the maintenance of the production potential of the orchards. Treatments may therefore be less targeted than in controlled conditions.

Secondly, orchard trees have to adapt constantly to the frequent changes of their surroundings induced by cultural operations (e.g., alternating of excess and limitation of water or nutrients due to periodic inputs) or climate. In 2019, for example, the temperatures were relatively colder than usual in April (mean temperature: 10.5 vs. 13.7 °C on average), which delayed shoot development and masked the differences in precocity between both varieties. This first period was then followed, between 24 June and 9 July, by a short, but intense, heat wave (maximal temperature 42 vs. 31 °C on average, mean temperature 28.4 vs. 24.5 °C on average) which again retarded shoot and fruit development. The tree response to these ongoing variations consists of phenological, developmental and compositional aspects, whose effects remain visible throughout the season. For instance, polyphenols are known to accumulate as a response to abiotic stress, which could partly explain the interannual differences [18,19]. Working in commercial orchards is therefore only possible if existing knowledge obtained in stable, controlled conditions helps to explain the plant response to infestation from surrounding changes. It is nevertheless an essential step in the development of innovative control strategies based on reducing tree susceptibility by the manipulation of overall tree conditions.

Thirdly, working on adult trees increases experimental complexity because of the necessary multiscale approach (tree, shoot and apex level). The biochemical determinations are necessarily performed on growing apices, rather than on phloem sap, which is not only difficult to collect, but whose composition and velocity are unstable over time and the diurnal cycle [20]. In contrast to phloem, apex concentrations reflect the prevailing conditions during apex formation. They act only as nutrient sinks, since leaves start to export C and other metabolites including N, only after their full expansion [21]. Indeed, apex composition has previously been successfully related to aphid development (e.g., [9] for total N), and could thus be considered as a reliable indicator of the conditions to which aphids are subjected during the development of their colonies.

### 4.2. Total N Concentration, a Marker of Plant Susceptibility?

Among the assessed variables, the only one which depended solely on shoot infestation status, being independent of year and varieties, was the N concentration. Thus, N could affect aphid development directly, or indirectly, through its impact on different plant metabolisms such as growth, development, or defence [8].

The direct effect of N was mediated by the amino acids, whose concentration is proportional to the one of total N [22], and which were involved in N transport [23,24] and, together with rubisco, N storage [22,25,26]. Since aphids are passive phloem feeders, their development is related to sap quality, whose concentration of amino acids is a major component. The amino acids which are considered as essential for aphid development (i.e., Thr, His, Phe, Val, Lys, Leu, Ile, Met, Trp) [10,27], being present in plants in only small amounts, must be synthetized by obligatory endosymbionts (e.g., *Buchnera aphidicola* for *M. persicae*), which use Glu, the main amino acid, as an N source [27,28]. However, according to Sauge et al. [9], the correlation between aphid development and leaf N status is positive until a threshold is reached around 3.5 N%DW, thereafter reversing and becoming negative. The cause of the inversion could not be precisely identified, even if the authors suspected that high N concentrations facilitated the induction of plant defence compounds. Our results confirm these assertions since the N concentration of the infested shoots was stable (i.e., around 3.3% DW), while those of the controls could be higher (i.e., above 3.5% DW in 2018) or lower (i.e., of 3 and 3.1% DW in 2019, respectively). The concentration of each amino acid was modified accordingly, but in most cases to an extent too small to be significant. The only exception was Conquise_2019, for which the differences where significant for two thirds of them.

### 4.3. Indirect Effects of N on Apex Composition

N also affects food quality indirectly, firstly through the N/C ratio of the phloem sap. The optimum value of the phloem amino acid/soluble sugar ratio is set between 0.1 and 0.2 [11,29]. Such values prevent aphids from ingesting excess C intake, and thus limit their production of honeydew.

Secondly, aphid development is negatively correlated with sap concentration in plant defence compounds. Among them, polyphenols are considered as markers of plant biotic and abiotic stresses. Several studies [30,31] have reported the existence of a competition between protein and phenolic synthesis, thus leading to a decrease in the polyphenol content on highly fertilized, and therefore, fast-growing plants [32,33]. Our results, particularly relating to the interannual differences in growth rates and polyphenol contents, could more likely be explained by the tree response to drought (leading to an earlier start of irrigation in 2019 than in 2018) and the succession of abnormal temperatures in 2019. Typically, cessation of growth precedes stomatal closure, thus leading to an accumulation of C and N since both elements are accumulated as long as the xylemic fluxes persist [34,35]. To limit cellular damage caused by water deficit, this C and N are invested in the synthesis of antioxidants, among which are phenols and tannins [36].

As a result, the concentration of polyphenols increased along with those of total N up to a threshold (Figure 3), which is in contradiction to the findings of Riipi et al. [32] and Herms [33]. This is probably related to the regional fertilization practices, which consist of boosting bud burst and early spring growth by high N inputs. Therefore, the N concentrations in the apices were, with a few exceptions, above the threshold set around 2.3% DW [37] which was limiting for shoot development. The competition for the N assimilates between growth and polyphenol metabolism was thus limited, but in spite of this, the shoot infestation status did not affect the polyphenol concentrations. Some compounds have been specifically identified as aphid feeding deterrents, among which are quercetins, kaempferols, flavonoids and phenolic acids [38,39]. A previous study under controlled conditions [8] also showed that, taken as a whole, they could be considered as good predictors of tree susceptibility. In open fields, in contrast, the instability of the surrounding conditions probably blurs the tree response to aphid infestation. Thus, trees react to transient stresses producing numerous secondary metabolites, including polyphenols, tannins, jasmonic and salicylic acids, whose effects on aphid development could be neutral, synergic, or antagonistic [12].

### 4.4. N Concentration and Shoot Growth and Development

In *Rocacea*, N availability affects shoot growth and development through (i) the number and position of the axillary buds which transform into long daughter axes, and (ii) the meristem activity, i.e., the GU production rates in those daughters and in the stem terminal (apical) meristem of the selected shoots [16,40]. Nonetheless, none of these processes was limited by N in the present study since the concentration in the apices was high enough not to limit growth [37].

Among the remaining candidates who could explain the observed differences, the most promising are (i) the intrinsic heterogeneity of the shoots within the tree canopy and (ii) the duration of the second growth stage. Indeed, shoots develop through alternating growth waves and rest periods, during which the nutrients are diverted to other organs, mainly roots and fruits [40,41]. The first stage corresponds to the apical and axillary bud burst and early spring growth, and its intensity depends on the amounts of C and N which can be mobilized from the stores [37]. The second stage starts after the trees become able to sustain their costs by current intake (i.e., by photosynthesis and root metabolism), which in a Mediterranean climate occurs usually at the end of April [42]. During this stage, the growth rates increase in proportion to the available nutrients [16], and the aphid populations, if any, increase rapidly. Shoot development is then again drastically slowed, approximatively one month before harvesting due to rapid fruit growth [43], or earlier if the water, C and/or N acquisition is reduced for any reason (deficit irrigation or high temperatures, among others). All shoots are however not equivalent and the duration of this second stage varies with shoot position within the canopy and sink strength, i.e., competition for nutrients and water between distant and neighbouring shoots [16,44].

In the present study, the plant pest interactions were not determined by the same developmental variables in both years. In 2019, infested and control shoots differed solely in their leaf expansion rates. These rates were also dramatically lower than in 2018, which suggests that the shoots were not growing over the entire reference period, and that growth was strongly limited during the June heat wave. The lower growth rate of the control shoots, compared to the infested ones, was accompanied by a higher N accumulation, which supports the idea that these shoots were more severely C limited, and therefore reduced or stopped their growth earlier.

In 2018, the differences were solely related to shoot shape since the infested shoots bore a higher number of leaves on their distal parts than the control ones. The daughter axes inserted on the upper growth units were longer on the infested shoots than on the control ones, meaning that they grew faster or longer. However, the mean leaf expansion rates (Table 3) were not affected, likely because the differences appeared at the end of the second growth stage, i.e., after growth cessation of the daughters inserted on the lower growth units. Indeed, shoot growth follows an acrotonic pattern [16]. Therefore, a growth rest affected first the daughters located on the basal growth units, then progressing upwards along the shoot stem, the daughters located on distal positions being the last to be limited. This pattern could nevertheless be disturbed by climatic events imposing a sudden growth decrease, as observed in 2019. In 2018, our results were in accordance with previous ones [5,8] linking infestation severity to shoot development.

### 4.5. From Shoot to Tree Level: Which Impacts Tree Vigour?

In the present study, shoot size, shape and composition were much less affected by infestation status than by variety and year, which could likely be related to the shoot selection process. Indeed, the control shoots were chosen so as to be as similar as possible to the infested ones, in order to assess the differences as the result of shoot main metabolisms prevailing during the development of the aphid populations, and not the consequence of pre-existent differences in shoot size and shape. Thus, selection was achieved after the first growth flush, when the differences in vigour and sink strength were already established [16] and was not representative of shoot heterogeneity within the tree canopy. Indeed, all selected shoots were vigorous and dominant ones, and therefore the less likely and slowest to be affected by restrictive treatments.

At tree level, infestation severity depended on the proportion of infested shoots, which varied with treatment (Table 3). Tree susceptibility to aphids was the lowest in the hn treatment, restricted in both water and N, thus enhancing the trophic competition between the shoots. Two descriptors of tree architecture, both playing a role in plant-aphid interactions [8,45], were modified by an N limitation: the number of vigorous shoots diminished and shoot heterogeneity within the canopy increased [20]. The differences between the two well-fertilized treatments were less marked, suggesting that tree susceptibility to aphids was mainly structured by N availability even if the interaction between water and N supply could also play a role. Indeed, the root N intake capacity was strongly reduced under water stress conditions [35], which thus increased the effect of N limitation. The effect of deficit irrigation on its own was also low because the deep loamy clay soil delayed the onset of water stress. In both 2018 and 2019, the ground water resources started to be limited only in June, as attested by the date of irrigation start (driven by the capacitive sensors). Therefore, the hN and HN trees experienced to the same conditions during the first stages of aphid infestation. Afterwards, aphid dispersion within the canopy seemed to favour the hN trees, specifically for Ivoire_2019. If deficit irrigation were applied earlier in the season, its effects could be more marked [3].

These results confirm previous ones relating tree susceptibility to aphids with the number of vigorous shoots, i.e., to tree vigour [5,7,8,45]. This assertion was also indirectly verified by the lower infestation observed (i) on Conquise (the less vigorous variety), as compared to Ivoire, and (ii) in 2019 (when shoot growth was smaller), as compared to 2018.

At tree level, the dominant aphid species changed between years, being less harmful in 2019 than in 2018. This shift, unexpected in our selected orchard, could possibly be due to the delay between the return flights of the different species, which started quite early (in September) for the green peach aphids in our region [46]. Therefore, the winter pruning undertaken in mid-October 2018, could have eliminated a significant part of the eggs and green peach aphid females, to the benefit of the alternative species. Indeed, it seems that the severe 2018 infestation did not affect significantly tree functioning and vigour. The shoot N content (results not shown) was 0.82 ± 0.029% DW, after leaf fall (i.e., on 5 November 2018), and such high values show that the autumnal storage was affected neither for N nor for C, to which it is correlated [42].

## 5. Conclusions

Our results confirmed that lowering simultaneously the water and N inputs reduces the severity of aphid infestation in commercial orchards. These practices could reasonably be implemented in commercial orchards since they do not alter fruit production: 150 and 121 fruits per tree in 2018 and 2019, respectively, with no effects of treatment on fruit size, i.e., on the commercial value (undetailed data). Our results are also consistent with previous ones obtained in young potted trees [3,8] in two aspects: whatever the culture conditions, aphid abundance increases with N supply and are lowest on the trees which are limited in both N and water. The N concentrations in the apices of the infested shoots are also similar in potted and orchard trees (i.e., around 3.2%), thus indicating this variable as a major determinant of shoot susceptibility. Adult and young potted trees differ greatly nonetheless when considering the effects of treatments and infestation status on shoot development and apex concentrations in polyphenols and NSC. The differences are related to the mean values and their variations with the shoot infestation status, being in most cases higher and more significant in young potted trees.

In orchards, the stress intensities during the infestation period are moderate, being buffered, firstly, by the soil water content and pluviometry which contribute greatly and in an unpredictable way to water supply, and secondly, by the high N inputs usually found at bud burst. The decrease in infestation severity induced by a limited and transient trophic stress could however not be sufficient, meaning that other alternatives have to be implemented at the same time to control efficiently aphid infestations. Among them, pruning, which could reduce the number of susceptible (vigorous) shoots by increasing their number, or changes in ground cover, have to be taken into consideration.

Our results were obtained from, and are therefore specific to, our localized conditions. To ensure their robustness and generality, they will now be confirmed across a wider range of genotypes and climatic conditions, by regular screenings performed over a large geographical area, which will probably be more informative than continuing our experiment beyond two years.

## Figures and Tables

**Table 1:** Water (irrigation) and N inputs per tree for each treatment and year. Notice that irrigation was limited to the period of water stress, and therefore started relatively late in spring: i.e., on 19 June 2018 and 1 June 2019 (HN: control, hN: deficit irrigation, hn: limited irrigation and N fertilization).

Year	Inputs Tree^−1^	HN (Control) Treatment	hN Treatment	hn Treatment
**2018**	**Nitrogen (g)**	84	84	56
	**Irrigation (L)**	22	14.7	14.7
**2019**	**Nitrogen (g)**	83	83	55.3
	**Irrigation (L)**	23	15.3	15.3

**Table 2:** Abbreviation of the polyphenols.

Polyphenols	Abbreviation
Chlorogenic acid (5-caffeoylquinic acid)	5CQA
Kaempferol-3-glucoside	K3Glu
3,5-dicaffeoylquinic acid	3,5-DiCQ
Kaempferol-3-galactoside	K3Gal
Quercetin-3-glucoside	Q3Glc
Hyperoside	Q3Gal
Neo-chlorogenic acid (3-caffeoylquinic acid)	3CQA
P-Coumaric acid	p_coum

**Table 3:** Aphid infestation at tree level according to treatment (HN: control, hN: deficit irrigation, hn: limited irrigation and N fertilization) and peach variety (Ivoire, Conquise). Classification of the trees (N = 54) regarding (a) the dominant aphid species they hosted, and (b) their maximum infestation severity estimated by a class of infestation (S0: no aphids, S1: <15% infested shoots, S2: between 15 and 50% of infested shoots, S3: >50% infested shoots).

		Ivoire			Conquise		
		HN	hN	hn	HN	hN	hn
(a) **Dominant aphid species**							
**2018**	No aphid	0	0	3	1	2	2
	Leaf-curl plum aphid	1	0	0	2	0	3
	Mealy plum aphid	0	1	1	1	2	0
	Green peach aphid	8	8	5	5	5	4
**2019**	No aphid	0	0	0	2	0	0
	Leaf-curl plum aphid	6	2	6	3	6	7
	Mealy plum aphid	1	0	1	2	2	2
	Green peach aphid	2	7	2	2	1	0
(b) **Infestation severity (maximum aphid abundance) of the dominant species**							
**2018**	S0	0	0	3	1	2	2
	S1	4	2	1	6	6	7
	S2	2	4	2	1	1	0
	S3	3	3	3	1	0	0
**2019**	S0	0	0	0	2	0	0
	S1	2	2	3	2	2	5
	S2	6	4	0	2	5	3
	S3	1	3	6	3	2	1

**Table 4:** Daily shoot growth rates (increase in number of expanded leaves day^−1^ parent shoot length in m^−1^) on the infested and control shoots (means and standard errors) according to treatment and peach variety (Ivoire, Conquise). The differences between control and infested shoots were significant (5% level, randomization tests based on the generation of 2500 orders) if coded with different letters. The growth rates were also significantly lower (randomization tests based on the generation of 2500 random orders) (i) in 2019 than in 2018 in all cases, (ii) for Conquise than for Ivoire except for control shoots in 2018. No differences could be found among treatments which were therefore grouped together.

	Ivoire		Conquise	
	Control	Infested	Control	Infested
**2018**	0.884 ^a^ ± 0.146	1.105 ^a^ ± 0.206	0.676 ^a^ ± 0.081	0.535 ^a^ ± 0.085
**2019**	0.351 ^a^ ± 0.107	0.614 ^b^ ± 0.089	0.121 ^a^ ± 0.031	0.367 ^b^ ± 0.073

## Data Availability

The data presented in this study are available in the article.
